# Incidence of emergency department presentations for traumatic brain injury in Indigenous and non-Indigenous residents aged 15–64 over the 9-year period 2007–2015 in North Queensland, Australia

**DOI:** 10.1186/s40621-018-0172-9

**Published:** 2018-11-12

**Authors:** Adrian Esterman, Fintan Thompson, Michelle Fitts, John Gilroy, Jennifer Fleming, Paul Maruff, Alan Clough, India Bohanna

**Affiliations:** 1 University of South Australia, Adelaide, South Australia 5000 Australia; 2 James Cook University, Cairns, QLD 4811 Australia; 3 University of Sydney, Sydney, NSW 2006 Australia; 4 The University of Queensland, Brisbane, QLD 4072 Australia; 5 University of Melbourne, Melbourne, VIC 3010 Australia

**Keywords:** Epidemiology, Head injury, Rural, Remote

## Abstract

**Background:**

Traumatic brain injury (TBI) is a leading cause of disability worldwide. Previous studies have shown that males have a higher incidence than females, and Indigenous populations have a higher rate than non-Indigenous. To date, no study has compared the incidence rate of TBI between Indigenous and non-Indigenous Australians for any cause. Here we add to this rather sparse literature.

**Methods:**

Retrospective analysis of data from North Queensland Emergency Departments between 2007 and 2015 using Australian Bureau of Statistics population estimates for North Queensland residents aged 15–64 years as denominator data. Outcome measures include incidence rate ratios (IRR) for TBI presentations by Indigenous status, age, sex, year of presentation, remoteness, and socio-economic indicator.

**Results:**

Overall incidence of TBI presentations per 100,000 population was 97.8. Indigenous people had an incidence of 166.4 compared to an incidence in the non-Indigenous population of 86.3, providing an IRR of 1.93 (95% CI 1.77–2.10; *p* < 0.001). Males were 2.29 (95% CI 2.12–2.48; *p* < 0.001) times more likely to present than females. Incidence increased with year of presentation only in the Indigenous male population.

**Conclusions:**

The greater burden of ED presentations for TBI in the Indigenous compared with the non-Indigenous population is of concern. Importantly, the need to provide quality services and support to people living with TBI in remote and very remote areas, and the major role of the new National Disability Insurance Scheme is discussed.

## Background

In Australia in 2008, injuries accounted for 9.3 per 1000 population of disability adjusted life years (Begg et al. 2008). In 2015–16, there were almost 7.5 million presentations to Australia’s public hospital emergency departments (ED), of which nearly one quarter were for an injury (Australian Institute of Health and Welfare 2016). Notably, injury rates for Indigenous Australians are twice that for non-Indigenous Australians (Jamieson et al. 2008), and those living in rural areas have 1.5 times the hospitalization rate for injury compared with those living in urban areas (Mitchell and Chong 2010).

Head injury is a common cause of ED presentations. Serious head injury or traumatic brain injury (TBI) can profoundly affect quality of life and lead to permanent impairment and disability (Jamieson et al. 2008).

TBI is a leading cause of disability in Australia (Helps et al. 2008). A systematic review of the incidence of TBI worldwide found a pooled incidence proportion of 295 (95% CI 274–317) per 100,000 population. The incidence proportion in males was significantly greater than in females (Nguyen et al. 2016).

A similar review focussing on Europe found an overall incidence of 262 (95% CI 185–339) per 100,000 population. They found the highest rates in people aged < 25 years and > 75 years, with a higher rate in males than females (Peeters et al. 2015). An American study found that Native Americans had greater rates of hospitalization for TBI than their non-Indigenous counterparts, and concluded that Indigenous peoples are over-represented in the TBI population (Rutland et al. 2005).

A previous study examined hospital admissions for head injury caused by assault in three Australian states. It found that Indigenous Australians were 21 times more likely to be admitted than non-Indigenous, with Indigenous women disproportionately represented (Jamieson et al. 2008). However, to date, no study has compared the incidence rate of TBI ED presentations for all causes of TBI between Indigenous and non-Indigenous Australians. Here we add to this rather sparse literature by describing the incidence of TBI based on ED presentations in North Queensland Australia. In addition, we compare rates by Indigenous status, age, sex, year, remoteness and socio-economic indicator.

## Methods

### ED information system data

**Fig. 1:**
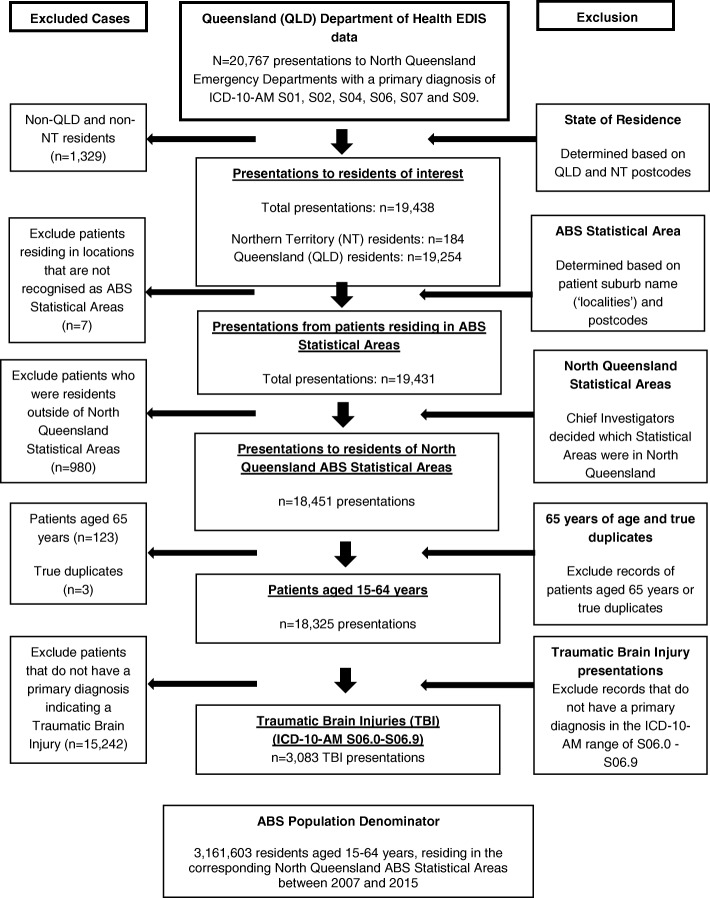
Flow chart showing the selection of ED presentations and the denominator population

This descriptive study analyzed ED data provided by the Queensland Government Department of Health. Individual records were first extracted for presentations with a primary diagnosis corresponding to a selection of ICD-10-AM head injury codes (ICD-10-AM code S01, S02, S04, S06, S07 and S09) (Fig. 1). For the purposes of the current study, final analyses were limited to records with a diagnosis of TBI (ICD-10-AM code S06 - Intracranial Injury). Records were de-identified and individuals assigned a unique identifier. Multiple records could have the same identifier, representing repeated presentations by an individual during the study period. Data were provided for presentations to the EDs of North Queensland hospitals between January 2007 and December 2015. This study region commences from approximately halfway up the Australian state of Queensland and extends north to the Torres Strait and west to the Gulf Country. Data were provided for patients aged 15–65 years at the time of presentation. This age range was selected since the study is part of a larger National Health and Medical Research Council (NHMRC) funded project on TBI in Indigenous people within this age group.

### Population level data for the North Queensland region

Patients not resident in Queensland or the Northern Territory were first excluded (see Fig. 1). Population data for the remaining presentations were obtained from the Australian Bureau of Statistics (ABS) (Australian Bureau of Statistics 2017a). For each presentation, the patient’s suburb of residence and postcode were used to identify a corresponding Queensland ABS Statistical Area 2 (SA2) region, using a correspondence file sourced from the ABS (Australian Bureau of Statistics 2017b). These SA2s were then aggregated to form nine larger Statistical Area 3 (SA3) regions relevant to the catchment areas of the hospitals within the North Queensland region, as determined by the authors. Annual calendar year population denominator data for the SA3s were obtained from the Statistical Services Branch of the Queensland Department of Health (Fig. 1). The Branch maintains Queensland population estimates by year, SA3 boundaries, Socio-Economic Indexes for Areas (SEIFA), Accessibility/Remoteness Index of Australia (ARIA), age, sex and Indigenous status. The data originate from several ABS data sources which are aggregated into a single file by a separate government department, the Queensland Government Statistician’s Office (Queensland Government Statistician’s office 2015).

The ABS SEIFA score was used to measure socio-economic disadvantage; a lower score reflecting more widespread and intensified disadvantage in the residents. The Accessibility and Remoteness Index of Australia (ARIA) geographic classification was used as a measure of remoteness of each patient’s residence. The patients in this study were resident in one of three ARIA levels; Outer Regional, Remote and Very Remote.

### Patient level data

Indigenous patients were Aboriginal, Torres Strait Islander or both. Records with missing Indigenous status or sex were only included in analysis of total rates. Similarly, records of patients aged 65 years at date of presentation were excluded from analyses as corresponding population data were only available in 5-year groups up to 64 years.

A subset of individuals in this study presented to the ED multiple times. There is inconsistency in the TBI literature regarding what constitutes a re-presentation for the same event. In emergency medicine research, 72 h is one interval used for tracking ED return visits, however, other timeframes (e.g. 48 h or 7 days) are also advocated (Shy et al. 2015). In the absence of a ‘best practice approach’, this study assumed that each presentation was a new episode. For comparison, analyses were undertaken after excluding presentations that occurred for the same individual within 168 h (i.e. 7 days) of his or her preceding presentation.

### Statistical analysis

Incidence rates and incidence rate ratios (IRR) are presented with 95% Confidence Intervals. As well as overall population data, data were aggregated by six study variables; age (5 levels), sex (2 levels), Indigenous status (2 levels), year of presentation (9 levels), remoteness (3 levels) and SEIFA (3 levels), to form 1620 records to enable regression modelling. After aggregation, each record contained the number of presentations and corresponding population for each combination of the six variables. Using this aggregated dataset, age and sex adjustment and trends in incidence rates were undertaken using Poisson regression with the relevant population data as the exposure variable. The Indigenous population is generally younger than the non-Indigenous population. To enable comparisons of ED presentation rates, crude rates were directly age-standardised to the 30 June 2001 Australian Standard Population maintained by the ABS (Australian Bureau of Statistics 2013). All analyses were undertaken using the Stata 14 software package (Stata Corp, College Station, Texas, USA).

## Results

Between 2007 and 2015 there were 3083 presentations for TBI in North Queensland EDs. The presentations were for 2811 unique individuals of whom 2592 (92.2%) presented once only, 177 (6.3%) presented twice (i.e. 354 presentations) and 42 (1.5%) presented on three or more occasions (i.e. 137 presentations) during the 9-year period.

### Traumatic brain injury (TBI)

Table 1 shows that the incidence of presentations for TBI (95% CI) per 100,000 population was 97.8 (94.4–101.3). Indigenous people were nearly twice as likely (IRR = 1.93, 95% CI 1.77–2.10, *p* < 0.001) to present with TBI than non-Indigenous people, and this result remained statistically significant after adjusting for age and sex (IRR = 1.70, 95% CI 1.56–1.85, *p* < 0.001). There was little change in the IRR when rates were age standardised (IRR = 1.86, 95% CI 1.70–2.03). Males were 2.29 (95% CI 2.12–2.48) times more likely to present with a TBI than females (*p* < 0.001). Based on a Poisson regression, the incidence of presentations overall declined linearly with age (*p* < 0.001).

**Table 1:** Rates of ED presentations for TBI in North Queensland (2007–2015) per 100,000 population

Selected variables	Presentations	Population at risk	Rate (100,000 population)	Incidence rate ratio (RR)	
			Rate (95% CIs)	RR (95% CIs)	P
All Traumatic Brain Injuries	3083	3,151,154	97.8 (94.4–101.3)		
Indigenous status					
Non-Indigenous	2375	2,753,418	86.3 (82.8–89.7)		
Indigenous	662	397,736	166.4 (153.8–179.1)	1.93 (1.77–2.10)	<0.001
Unknown	46				
Sex					
Female	918	1,553,724	59.1 (55.3–62.9)		
Male	2165	1,597,430	135.5 (129.8–141.2)	2.29 (2.12–2.48)	<0.001
Age categories					
15–24	1187	655,073	181.2 (170.9–191.5)		
25–34	617	655,420	94.1 (86.7–101.6)	0.52 (0.47–0.57)	<0.001
35–44	505	671,141	75.2 (68.7–81.8)	0.42 (0.37–0.46)	<0.001
45–54	432	649,583	66.5 (60.2–72.8)	0.37 (0.33–0.41)	<0.001
55–64	342	519,937	65.8 (58.8–72.7)	0.36 (0.32–0.41)	<0.001
Year of ED presentation					
2007	307	328,306	93.5 (83.0–104.0)		
2008	338	337,217	100.2 (89.5–110.9)	1.07 (0.92–1.25)	0.400
2009	343	344,501	99.6 (89.0–110.1)	1.06 (0.91–1.24)	0.448
2010	322	349,363	92.2 (82.1–102.2)	0.99 (0.84–1.15)	0.888
2011	303	353,502	85.7 (76.1–95.4)	0.92 (0.78–1.07)	0.301
2012	285	356,850	79.9 (70.6–89.1)	0.85 (0.73–1.00)	0.060
2013	347	359,589	96.5 (86.3–106.7)	1.03 (0.89–1.20)	0.717
2014	399	360,815	110.6 (99.7–121.4)	1.18 (1.02–1.37)	0.030
2015	439	361,011	121.6 (110.2–133.0)	1.30 (1.12–1.50)	0<.001
Remoteness (ABS ARIA classification)					
3. Outer Regional	2491	2,738,822	91.0 (87.4–94.5)		
4. Remote	465	252,515	184.1 (167.4–200.9)	2.02 (1.83–2.24)	<0.001
5. Very Remote	127	159,817	79.5 (65.6–93.3)	0.87 (0.73–1.04)	0.149
Socio-Economic Indexes for Areas (SEIFA) decile					
1–2	1214	757,007	160.4 (151.3–169.4)		
3–4	883	1,049,982	84.1 (78.5–89.6)	0.52 (0.48–0.57)	<0.001
5–6	349	563,371	61.9 (55.4–68.4)	0.39 (0.34–0.44)	<0.001
7–8	467	524,536	89.0 (81.0–97.1)	0.56 (0.50–0.62)	<0.001
9–10	170	256,258	66.3 (56.4–76.3)	0.41 (0.35–0.49)	0.000

**Fig. 2:**
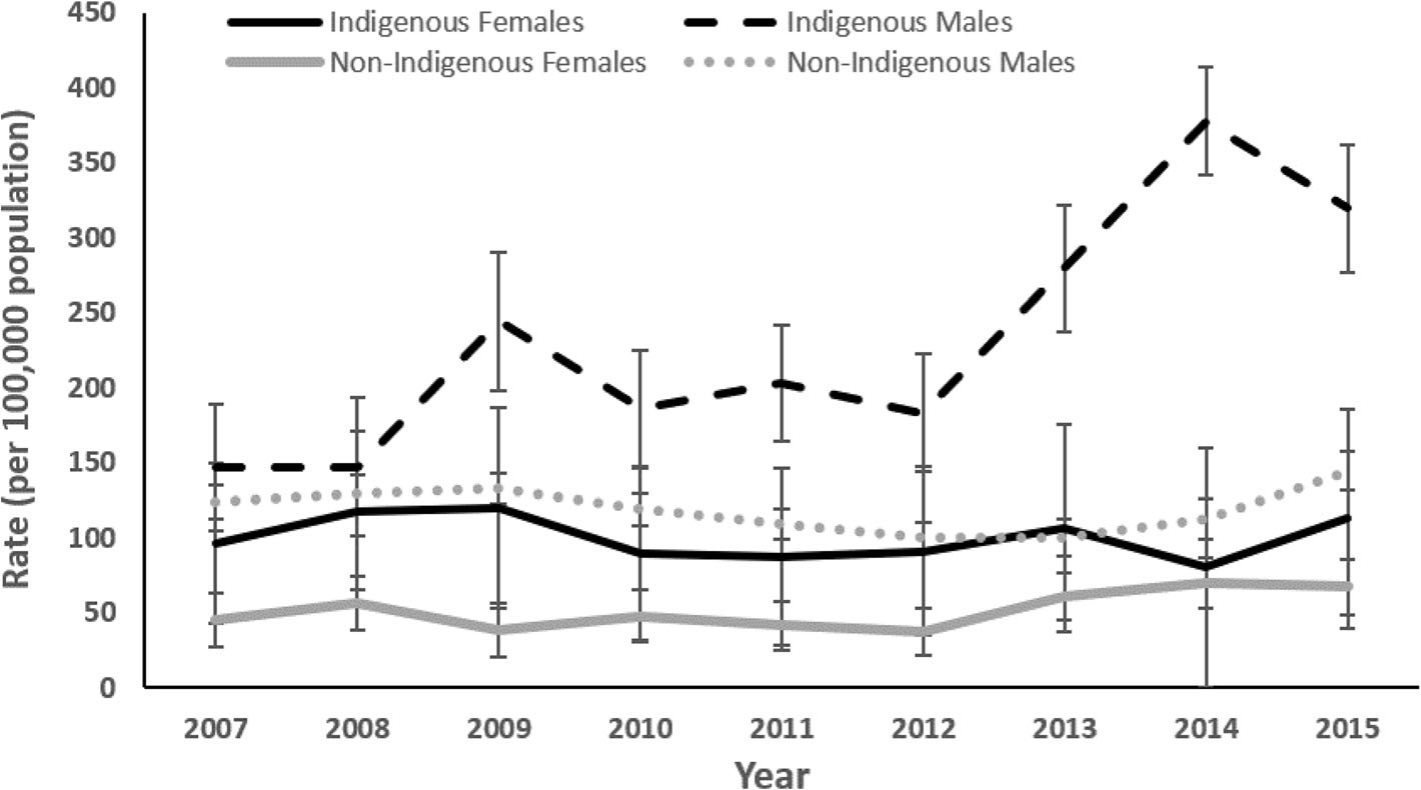
Annual rates of ED presentations for Traumatic Brain Injury (ICD-10-AM S06 Intracranial Injury range), by sex and Indigenous status, North Queensland (2007–2015) per 100,000 population

Figure 2 shows that the incidence of TBI was stable over the time period of the study for all groups except Indigenous males, where an increasing linear trend was seen (Poisson regression; *p* < 0.001).

Overall, there was not a consistent pattern of TBI rate by ARIA status. However, when stratified by Indigenous status, Indigenous people residing in a Remote area had a significantly increased rate of TBI compared with those living in an Outer Regional area (IRR = 1.81, 95% CI 1.53–2.15, *p* < 0.001), whereas those living in a Very Remote area had a much lower rate (IRR = 0.41, 95% CI 0.32–0.54, *p* < 0.001). For non-Indigenous people, those living in Remote areas still had an increased risk compared with their counterparts in Outer Regional areas (IRR = 1.65, 95% CI 1.45–1.88, *p* < 0.001), however, living in a Very Remote area was no longer protective (IRR = 1.03, 95% CI 0.80–1.34, *p* = 0.799).

A formal comparison of SEIFA scores 1 or 2 versus 3 or more found that those residing in areas with SEIFA scores of 1 or 2, had 2.02 (95% CI 1.88–2.18, p < 0.001) times the rate of TBI presentations. Separate regression models for Indigenous and non-Indigenous populations found that the risk attached to socioeconomic disadvantage was greater for non-Indigenous populations (IRR = 1.95, 95% CI 1.79–2.13, p < 0.001), compared with Indigenous populations (IRR = 1.41, 95% CI 1.21–1.66, *p* < 0.001).

## Discussion

This study examined presentations for TBI in North Queensland EDs between 2007 and 2015. Indigenous people were twice as likely to present to an ED for TBI than non-Indigenous people, males were at higher risk than females and those living in socio-economically deprived areas were at higher risk than those living in wealthier areas.

This is in contrast to a previous Australian study that found quite different risk estimates (Jamieson et al. 2008). This study found that Indigenous Australians were 21 times more likely to be admitted to hospital for a head injury caused by assault, compared with non-Indigenous Australians. In particular, Indigenous women were greatly over represented. However, the population included in the Jamieson et al. paper was quite different from the population in this study. It was a study of hospital admissions, not ED presentations; it included all head injury codes (S00 – S009) and only those attributed to ‘assault’, whereas our study focused on the TBI code (S06) alone, irrespective of external cause attributed. The Jamieson et al. study also included all public and private hospitals in three Australian states, which would primarily have reflected metropolitan areas. In contrast, our study population was either very remote, remote, or outer regional. Finally, the 2008 paper was for all ages compared with our narrower age range.

Of interest in our data are the patterns of incidence rates found in the Indigenous compared with the non-Indigenous population. Whilst rates of presentations for TBI were stable in other population groups, those for the Indigenous male population appeared to have been steadily increasing. In 2007, Queensland implemented a policy prohibiting alcohol sales, possession and consumption in several remote Indigenous localities in north Queensland on the back of laws first implemented in 2002, limiting the possession of alcohol in community areas declared restricted (Margolis et al. 2011, West et al. 2017). The prospect that a rising trend in TBI rates is apparent despite alcohol restrictions, particularly among Indigenous males (Fig. 2), warrants further analysis to examine the possible differing roles of alcohol in brain injury for Indigenous males compared with other segments of the population.

We also found that Indigenous people were at higher risk if they lived in a Remote area compared with an Outer regional area, but at a lower risk if they lived in a Very remote area, and this latter pattern was not seen in non-Indigenous people. The region of the current study includes suburbs (closer to hospitals) in or near urban centres, where the local population is comprised largely of people who have migrated there from remote regions. There are strong similarities in social circumstances in the populations in these suburbs and the more remote and very remote communities located far from a hospital emergency department. The ARIA levels reflect the physical remoteness of a locality and likely do not capture the social characteristics of these areas and service accessibility. Non-Indigenous people living in socio-economically deprived areas had higher rates than those living in wealthier areas, but the pattern was less clear in the Indigenous population. The question then arises as to whether Indigeneity per se is a risk factor for TBI, or whether it is mediated through socio-economic disadvantage, remoteness or other factors not included in our data. While we can speculate that the social pathologies underpinning the causal mechanisms of TBI are differently configured in the two groups, further detailed research is required to investigate this novel finding across these continua.

Australian Indigenous people experience significant culturally related hardships and disadvantages in accessing disability services (Gilroy et al. 2016).

Since July 2016, the Australian Government has progressively introduced the National Disability Insurance Scheme (NDIS). This scheme provides support to people under the age of 65 who have a permanent impairment, including those with a brain injury (National Disability Insurance Agency 2016). Apart from providing income support, the NDIS provides access to information about available support options and referral to relevant disability services. It aims to help build individual capacity through assistance with diagnosis advice, peer support and skills development. Our findings show high rates of TBI among the Indigenous population across all geographic and social strata, including disadvantaged and/or remote communities. For those eligible Indigenous people, the NDIS plans will need to be tailored to the cultural, financial, and environmental needs to ensure equitable access to services that enhance their economic and social participation, and to enable them to remain on their traditional homelands. Such injuries have a drastic impact on cultural and social responsibilities and practice in Indigenous communities. The Australian government has funded many NDIS strategies to support Aboriginal and Torres Strait islander communities, including community-controlled organisations. This also includes ensuring that the NDIS planning process is culturally responsive and respectful.

Finally, the National Disability Insurance Agency, the government department responsible for the NDIS, could collaborate with the hospital systems in North Queensland to better prepare NDIS plans for people with TBI that will foster timely rehabilitation from the hospital and into the community.

Notably, we were reliant on the accuracy of routinely collected ICD-10-AM coded ED data. This classification system is intended for administrative purposes and has a limited application in epidemiological research (Roozenbeck et al. 2013). As a result, the rates and modelling we report should be interpreted with caution. Anecdotal reports from health professionals interviewed as part of the larger NHMRC project suggest there has been increased reporting and recognition of TBI in recent years. While this is unlikely to account for the entire increase in rates noted in our study, it is possible increased awareness of TBI may have made some contribution. Our upper age cut off age of 64 years meant that we could not demonstrate an expected peak in presentations in older ages and does make it more difficult to compare incidence rates with other studies. The inability to distinguish repeat presentations within a short time-frame arising from the same or new injury means that the incidence rate presented here may be inflated. However, excluding presentations that occurred within 168 h (i.e. 7 days) did not result in a substantial decrease in incidence rates or the overall Indigenous to non-Indigenous IRR (results not tabled).

## Conclusions

Like many previous studies, we found that males were more likely than females to present at an ED with a TBI. We also found that Indigenous people were twice as likely to present as non-Indigenous. Of concern, is an apparent increasing rate of TBI presentations in Indigenous males in this region. This is despite alcohol restrictions being in place in many of the remote communities which, as evidence shows, reduced violence and serious injury generally in these communities (Margolis et al. 2011). However, recent evidence suggests that the positive effects of these restrictions are reversing (Clough et al. 2018), making it essential to examine the possible impacts on TBI rates in more detail. We also found interesting patterns in incidence rates that clearly need further research. For example, Indigenous people living in very remote areas having lower rates than other groups. This may be an issue of limited access to relevant treatment services locally in these remote settings and to the difficulties of transport to suitable treatment services in the regional centres. This also warrants further examination to address this inequity due to isolation.

The primary government support service for those with TBI is the NDIS. The NDIS is working closely with Indigenous communities to develop suitably culturally sensitive support services for Indigenous people with a disability, including those living in remote areas.

## Abbreviations

ABS: Australian Bureau of Statistics
AIHW: Australian Institute of Health and Welfare
ARIA: Accessibility and Remoteness Index of Australia
ED: Emergency Department
NDIS: Australian National Disability Insurance Scheme
NHMRC: Australian National Health and Medical Research Council
SEIFA: Socio-Economic Indexes For Areas
TBI: Traumatic Brain Injury
